# Quantitative Characterization of Tensile Strength for Carburized Materials Using a Novel Composite Strength Method

**DOI:** 10.3390/ma18225205

**Published:** 2025-11-17

**Authors:** Hongjun Wang, Yanding Guo, Shiqi Chen, Xuming Zha, Zejie Li, Zhilong Xu

**Affiliations:** 1 College of Marine Equipment and Mechanical Engineering, Jimei University, Xiamen 361021, China; 2 State Key Laboratory of Intelligent Manufacturing Equipment and Technology, Huazhong University of Science and Technology, Wuhan 430074, China

**Keywords:** quantitative characterization, tensile strength, Voigt-related multilayer method, carburized specimen, experimental validation

## Abstract

Carburizing is a cost-effective surface hardening process that significantly enhances the strength of components and is widely used in critical parts such as main transmission gears and cams. However, research on mathematical models for characterizing the strength of carburized layers remains underdeveloped, limiting the accurate assessment of the service strength of carburized workpieces. To address this issue, this study focuses on 20CrNiMo steel and proposes a novel composite strength method for the quantitative prediction of the tensile strength of carburized specimens. By establishing a continuous mapping function between microhardness measurements and tensile strength distribution, and developing an equivalent strength mathematical model for non-uniform carburized gradient structures, the proposed method successfully predicts the strength of specimens under different carburizing depths. This approach represents a departure from the conventional Voigt-based paradigm, which relies on layered discretization of the carburized zone for approximate estimation, leading to significant improvements in both predictive efficiency and accuracy. Based on experimental data, the proposed method achieves a reduction in prediction errors of over 41.8% compared to traditional multilayer methods. In summary, this study not only provides a reliable and efficient computational tool for the quantitative characterization of carburized steels, but also demonstrates strong potential for application in the strength assessment and design optimization of critical engineering components.

## 1. Introduction

With the continuous advancement of industry, conventional manufacturing technologies have gradually become inadequate to meet the increasingly demanding performance requirements for components in specialized fields such as aerospace [1], marine engineering [2], and the nuclear industry [3]. Currently, the primary process methods for improving the properties of metallic materials include alloying treatment [4], surface deformation strengthening [5,6,7,8,9], and heat treatment [10,11,12,13]. While alloying treatment can enhance material performance, it is impossible to meet all service requirements of metal components using just a single alloying element. Surface deformation strengthening, although beneficial, typically produces only shallow affected layers [14], resulting in limited improvements in material properties. In contrast, carburizing heat treatment has become a powerful engineering solution for components such as main drive gears [15] and cams [16], significantly enhancing their hardness, strength, and fatigue resistance. This widespread adoption is attributed to its high process reliability [17] and superior efficiency [18].

Extensive research has investigated the effects of carburization on the mechanical properties of steels. Shi et al. [19] employed cyclic carburizing to achieve a high-hardness, dense, and thick hardened layer in 17CrNiMo6 heavy-duty gear steel. Touati et al. [20] revealed that among the three key carburizing parameters—carbon potential, carburizing temperature, and carburizing processing time—the processing time emerged as the dominant factor affecting hardness in low-alloy steels, while the carburizing temperature primarily governed the hardened case depth. Mathews et al. [21] characterized the microstructural evolution during carburizing, identifying distinct phase distributions: the high-carbon surface region consisted predominantly of ferrite and cementite, while the low-carbon core region exhibited a martensitic structure with eutectoid ferrite. Mo et al. [22] demonstrated that under identical experimental conditions, 20CrMnTi gear steel subjected to carburizing followed by normalizing at 940 °C exhibited both smaller average grain size and more uniform grain size distribution compared to treatment at 800 °C. Wang et al. [23] revealed that 18CrNiMo7-6 steel achieved approximately 33% grain refinement in the carburized layer after treatment, with solid solution strengthening by carbon atoms and grain boundary strengthening identified as the predominant strengthening mechanisms. Farivar et al. [24] discovered that increased bainite formation during carburization significantly enhanced the impact toughness of carburized steel components. This improvement was primarily attributed to the intrinsic structure of bainite grain boundaries, which effectively promotes crack deflection during propagation. Most previous works primarily relied on qualitative analysis based on experimental observations, while quantitative characterization of the carburized layer remains insufficient. This limitation arises because the carburized layer exhibits a continuously graded distribution of microstructure and properties, essentially making it a functionally graded material, for which accurate equivalence of strength and fatigue life is highly challenging. In contrast, for homogeneous materials and conventional composite materials, many scholars have already developed mature approaches to characterize and model their mechanical strength.

**Figure 1:**
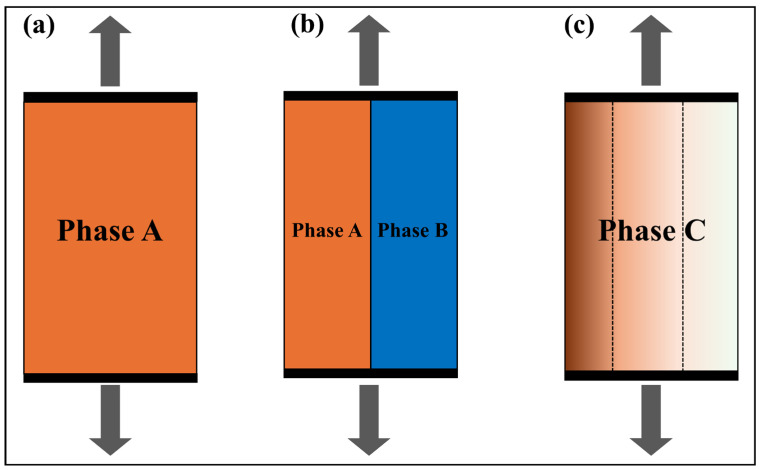
Schematic diagrams of different materials under iso-strain conditions: (**a**) Homogeneous material; (**b**) Voigt model for a two-phase composite; (**c**) Multilayer method for a functionally graded material.

For homogeneous materials, as illustrated in Figure 1a, empirical formulas are commonly employed for quantitative characterization, such as establishing the mapping relationship between hardness and strength [25,26]. In the case of a simple composite material system consisting of two constituent matrices [27], the classical Voigt [28] or Reuss [29] model can be selected based on different mechanical assumptions. The Voigt model is based on the iso-strain assumption, implying that each component (or layer) experiences identical strain, whereas the Reuss model relies on the iso-stress assumption, whereby each constituent (or layer) is subject to uniform stress. As one of the earliest mixing-rule models for composite materials, the Voigt model has been widely used in predicting the mechanical behavior of composites [30,31], its schematic representation of this model is provided in Figure 1b.

For functionally graded materials, researchers have developed various methods for characterizing their mechanical properties [32,33,34,35]. As a typical type of functionally graded materials, the quantitative characterization of carburized layers still faces challenges, which has motivated several studies in recent years dedicated to the development of accurate models. Zhao et al. [36] developed an innovative method for measuring the elastic modulus of the carburized layer, revealing that the modulus remains essentially constant and shows negligible dependence on carbon content or depth. Zhang et al. [37] investigated the fatigue behavior of the carburized layer using a layer-wise sampling approach, and observed that the slope of the S-N curve gradually decreases with increasing carburized layer thickness. Similarly employing a layered analysis method, Wang et al. [38] reported that the residual compressive stress initially increases and then decreases with carburizing depth, eventually stabilizing in the substrate region. Electrochemical tests further indicated that a reduction in residual compressive stress leads to an increase in both electrochemical impedance and corrosion potential. Čular et al. [39,40] proposed a multi-layer discrete computational model in which the gear root region is divided into multiple layers, each assigned constant hardness and residual stress values, to predict the bending fatigue failure location and service life of surface hardened spur gears.

In summary, current research primarily focuses on two aspects: first, the qualitative analysis of the mechanisms through which carburizing enhances material properties; and second, the quantitative characterization based on the multilayer method. By using this layered method, the distribution of mechanical properties along the depth of the carburized layer can be obtained. Essentially, the multilayer method can be regarded as an extension of the Voigt model to functionally graded structures, as illustrated in Figure 1c. This approach essentially approximates the continuous functional gradient by discretizing it into segments with distinct material properties. As an approximation method, it inherently lacks guaranteed predictive accuracy. Theoretically, increasing the number of layers improves the prediction accuracy, bringing it closer to the actual behavior. However, it also leads to a significant rise in experimental and computational costs. Therefore, there is an urgent need to develop an equivalent model that balances predictive accuracy and computational efficiency for the strength prediction of carburized materials.

This study investigates the widely used low-carbon alloy steel 20CrNiMo and proposes an innovative composite strength method for the quantitative prediction of the tensile strength of carburized specimens. The main contributions of this work include establishing a mapping relationship between discrete hardness measurements and continuous strength gradients, developing an equivalent strength prediction model based on continuous gradient strength distribution, and experimentally validating the improved predictive accuracy and reliability of the proposed method compared to conventional layered models. The results demonstrate that this approach not only enables high-precision quantitative characterization of the mechanical properties of the carburized layer but also offers simplicity in operation and ease of engineering application, providing an effective theoretical tool and practical means for strength assessment of carburized components.

The structure of this paper is organized as follows: Section 2 introduces the fundamental principles of the novel composite strength method and derives a quantitative predictive model for the strength of carburized specimens. Section 3 provides a detailed description of the experimental methodology and processing parameters. Section 4 focuses on model validation and results analysis, evaluating the predictive accuracy through comparison between theoretical predictions and experimental measurements. A comparative analysis between the proposed method and the Voigt-related multilayer method is also included. The final section presents concluding remarks. Through this systematic approach, the study establishes a novel composite strength method specifically applicable to carburized specimens for tensile strength prediction and demonstrates its engineering applicability.

## 2. Quantitative Characterization of Carburized Specimens

The core objective of this paper is to propose and validate a composite strength method applicable to carburized steels. To achieve this fundamental goal, we deliberately selected standard round tensile specimens conforming to national standards. The primary rationale is that current research in this field remains scarce without established theories available for reference, while round bar specimens represent the most fundamental and standardized form for characterizing tensile properties. This approach isolates the material strength gradient introduced by carburizing from the complex geometries of actual components, allowing it to be fully incorporated into the model. Consequently, standard round bar tensile specimens conforming to national standards were selected as the subject of analysis.

**Figure 2:**
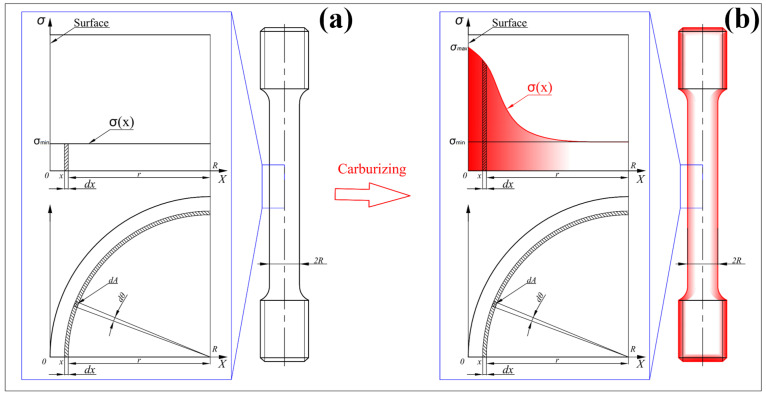
Schematic diagram of the strength distribution of the surface layer of the tensile specimen: (**a**) Before carburizing; (**b**) After carburizing.

For the non-carburized specimen, the strength exhibits a uniform distribution (Figure 2a), and the stress equation is expressed as:

(1) $$\bar{\sigma }=\frac{F}{A}=\frac{dF}{dA},$$

where *A* represents the cross-sectional area of the cylinder (mm^2^), *F* denotes the applied load (N), *dA* is the differential area of the infinitesimal ring, and *dF* stands for the equivalent force acting on the differential element *dA*.

However, for the carburized specimen, the strength exhibits a non-uniform distribution along the radial direction, as shown in Figure 2b. It should be noted that the carburized layer exhibits a continuous gradient structure, with strength gradually decreasing from the high-strength surface to the low-strength core. During tensile loading, the surface may still remain in the elastic stage while the core has already yielded and entered the plastic stage. This coexistence of elastic and plastic states induces complex mechanical interactions between the surface and core regions. And the underlying mechanisms are not yet fully understood. Therefore, at the macroscopic equivalent level, this study adopts the fundamental concept of the Voigt model, setting aside an in-depth exploration of the intricate internal interactions, and instead employs the iso-strain assumption to predict the overall material performance. Under this condition, the loading on each infinitesimal element of the specimen satisfies the following relationship with its local strength:

(2) $$dF=\sigma_{\left(A\right)}dA,$$

where *σ*_(*A*)_ is the distribution function of strength on the differential area.

By combining Equation (1) with Equation (2), we obtain the equivalent strength on the cross-section of the carburized round bar tensile specimen as:

(3) $$\bar{\sigma }=\frac{\int_{A}\sigma_{\left(r\right)}dA}{A}=\frac{\int_{0}^{2\pi }\int_{0}^{R}\sigma_{\left(r\right)}rdrd\theta }{\pi R^{2}}=\frac{2\int_{0}^{R}\sigma_{\left(r\right)}rdr}{R^{2}},$$

where *r* is the distance from the center of the circle (mm), *R* is the cross-sectional radius (mm). *σ*_(*r*)_ is the distribution function of strength along the radius within the nominal length.

Let the carburization depth (measured from the specimen surface toward the core) be denoted as *x* (mm), which satisfies *x* = *R* − *r*. Using integration by substitution [41,42], Equation (3) can be rewritten as:

(4) $$\bar{\sigma }=\frac{2\int_{0}^{R}\sigma_{\left(r\right)}rdr}{R^{2}}=\frac{2\int_{R}^{0}\sigma_{\left(x\right)}(R-x)d(R-x)}{R^{2}}=\frac{2\int_{0}^{R}\sigma_{\left(x\right)}(R-x)dx}{R^{2}}.$$

It should be emphasized that although the carburized layer is confined to a certain depth beneath the surface, the integration with respect to x must be performed over the entire range from the surface (*x* = 0) to the center (*x* = *R*) in order to calculate the equivalent strength of the entire cross-section.

The strength and hardness of steel both reflect the ability of the material to resist plastic deformation. The plastic deformation mechanism of steel is mainly dislocation slip [43,44], and the factors hindering dislocation movement (such as grain boundaries, second-phase particles, solid-solution atoms, etc.) are synchronous to the enhancement of strength and hardness, so that the same microscopic deformation mechanism makes the two positively proportional to each other, can be expressed as follows [26,45,46]:

(5) $$\sigma_{b}=(2.876\sim 3.734)\mathrm{HV}-(60\sim 200).$$

In this study, the slope coefficient was set to “3” [46], and the mean microhardness (430 HV) of the non-carburized specimen (NC) along with the tensile strength (1132.8 MPa, detailed data sources in Section 4.1 and Section 4.3) were substituted into the relationship σ = 3 HV + *b*. Through back-calculation, the intercept *b* was determined to be approximately −160, thereby completing the material-specific calibration of this empirical relationship. Consequently, we defined the equation as:

(6) $$\sigma_{b}=3\mathrm{HV}-160,$$

where *σ_b_* is the tensile strength (MPa), HV is the Vickers hardness. It should be noted that for other functionally graded materials or different microstructures, if similar hardness-strength correlations, which may have different functional forms or parameters, have been explicitly reported in the literature, the method can be extended in principle. Conversely, if the correspondence between hardness and strength is missing, the model should be applied with caution.

Therefore, to evaluate *σ*_(*x*)_, it is necessary to obtain the gradient data describing the strength distribution of the carburized layer along the depth direction. In this study, the measured microhardness gradient profile is incorporated into Equation (6), and a Taylor series expansion [47,48] is applied to approximate the carburization depth–strength relationship. The resulting expression is given as follows:

(7) $$\sigma_{\left(x\right)}=a_{0}+a_{1}x+a_{2}x^{2}+\ldots +a_{n}x^{n},$$

where *a_n_* is the order coefficient (*n* = 0, 1, 2…).

Substituting Equation (7) into Equation (4) gives the equivalent strength:

(8) $$\bar{\sigma }=\frac{2\int_{R}^{0}-a_{0}R+(a_{0}-a_{1}R)x+(a_{1}-a_{2}R)x^{2}+\ldots +(a_{n-1}-a_{n}R)x^{n}+a_{n}x^{n+1}dx}{R^{2}}.$$

Further organizing Equation (8), it follows that:

(9) $$\bar{\sigma }=2a_{0}+(a_{1}R-a_{0})+\frac{2}{3}(a_{2}R-a_{1})R+\ldots +\frac{2(a_{n}R-a_{n-1})R^{n-1}}{\left(n+1\right)}-\frac{2a_{n}R^{n}}{n+2},$$

and then collation yields:

(10) $$\bar{\sigma }=a_{0}+\frac{1}{3}a_{1}R+\frac{1}{6}a_{2}R^{2}+\frac{1}{10}a_{3}R^{3}\ldots +\frac{2}{n(n+1)}a_{n-1}R^{n-1}+\frac{2}{\left(n+1)(n+2\right)}a_{n}R^{n}.$$

This equation represents the final form of the composite strength method. Therefore, the core parameter of the composite strength method is the carburized layer gradient microhardness data without the need for static tensile testing.

When *σ*_(*x*)_ is a constant and equal to *a*_0_, $\bar{\sigma }$ also equals *a*_0_. In this case, *a*_0_ represents the tensile strength of the homogeneous, non-carburized 20CrNiMo base material. In this scenario, Equation (10) simplifies to the solution for a homogeneous material.

If the carburized layer is discretized using a Voigt-related multilayer method, Equation (4) can be reformulated in discrete form as follows:

(11) $$\bar{\sigma }=\frac{2\sum_{i=1}^{c}\sigma_{i}\int_{x_{i-1}}^{x_{i}}(R-x)dx}{R^{2}}\;(c\;\mathrm{is}\;a\;\mathrm{constant}).$$

In this context, the Voigt model emerges as a special case of Equation (4).

For a non-uniform *σ*_(*x*)_, the function is expanded into a Taylor series using the method described above. Consequently, $\bar{\sigma }$ can be represented as an explicit function of the series coefficients, as shown in Equation (10).

## 3. Experiments

### 3.1. Material

**Figure 3:**
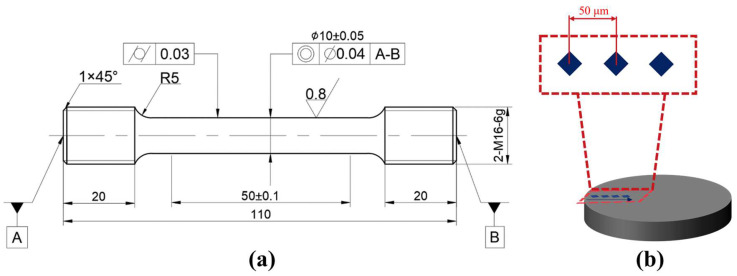
Test specimens of 20CrNiMo steel: (**a**) Tensile test specimen; (**b**) Microhardness test specimen.

The experimental material employed in this study was 20CrNiMo alloy steel, with its chemical composition detailed in Table 1. Dumbbell-shaped specimens were machined for carburizing treatment and tensile testing, conforming to the dimensional specifications of the Chinese standard GB/T 228.1-2021 [49]. The specimen geometry is illustrated in Figure 3a, with critical dimensions annotated.

### 3.2. Heat Treatment

The specimens were subjected to two distinct heat treatment groups: a conventional quenching and tempering process, and two variants of carburizing followed by quenching and tempering. The quenching and tempering process (designated as specimen NC) involved austenitization at 820 °C for 45 min, oil quenching, and tempering at 200 °C for 120 min. The carburizing processes were differentiated by their carburization parameters: specimen C1 underwent boost-stage carburization at 930 °C for 120 min at a carbon potential of 1.1%, followed by a 180 min diffusion stage at a carbon potential of 0.8%, specimen C2 experienced the same boost-stage carburization conditions but for 60 min, followed by a 120 min diffusion stage. Both C1 and C2 subsequently underwent identical post-carburization thermal treatment: austenitization at 820 °C for 45 min, oil quenching, and tempering at 200 °C for 120 min. The critical distinction between the NC and carburizing groups was the application of carburization, while the variation between C1 and C2 lay exclusively in the duration of the boost and diffusion stages during carburization.

### 3.3. Microhardness and Tensile Test

After wire cutting the carburized tensile specimen, the microhardness profile from the surface to the core along the cross-sectional depth (at 50 μm intervals) was measured using a FALCON-500 hardness tester (INNOVATEST Europe BV, Maastricht, The Netherlands), the schematic diagram is shown in Figure 3b.

The MTS microcomputer-controlled electronic universal testing machine (MTS Systems Corporation, Eden Prairie, MN, USA) was used for quenching–tempering and carburizing-quenching-tempering two groups of specimens to carry out tensile experiments (displacement rate of 0.05 mm/s).

## 4. Results and Discussion

### 4.1. Results of Microhardness

**Figure 4:**
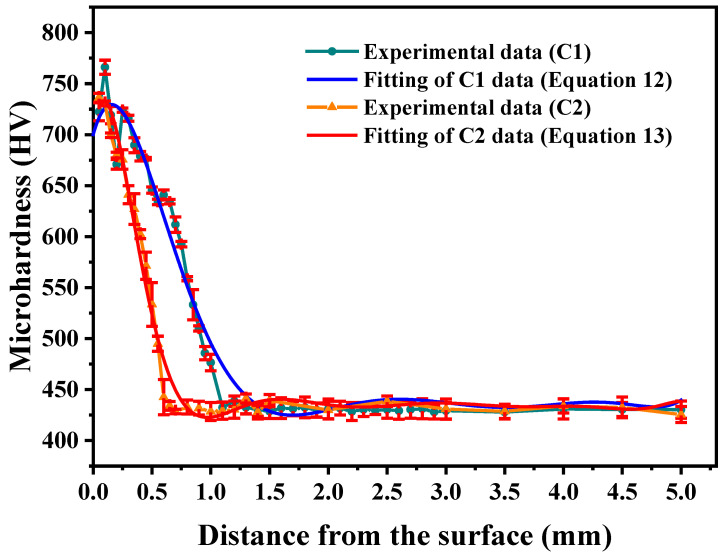
Microhardness distribution of data for the carburized layer.

Figure 4 presents the experimentally measured microhardness profile of the carburized layer along with its corresponding Taylor model fitting. In the Figure, the blue solid line represents a polynomial fit of the microhardness measurements for specimen C1 along the depth direction, expressed by the following equation:

(12) $$\begin{array}{l} \mathrm{HV}(x)=1.165x^{7}-23.137x^{6}+184.730x^{5}-752.270x^{4}+1615.115x^{3}\\ \;\;\;\;-1637.253x^{2}+407.042x+700.156. \end{array}$$

The determination of the Taylor series order for Equation (12) is described in Appendix A. The data in Figure 4 show that the cross-sectional hardness profile of specimen C1 demonstrates a progressive decline from the surface to the core, attaining substrate hardness levels at a depth of 1.1 mm. The carburized surface layer of C1 achieves a peak hardness of 768 HV, while its core region maintains a base hardness of 430 HV.

As illustrated by the red solid line in Figure 4, the polynomial fitting results representing the microhardness distribution along the depth direction of specimen C2 are presented, with the corresponding polynomial expression given by the following equation:

(13) $$\begin{array}{l} \mathrm{HV}(x)=-0.268x^{1}{⁠}^{0}+7.514x^{9}-90.870x^{8}+620.475x^{7}-2622.669x^{6}\\ \;\;\;\;+7066.043x^{5}-12003.211x^{4}+12150.308x^{3}\\ \;\;\;\;-6303.282x^{2}+905.120x+693.770. \end{array}$$

Notably, specimen C2 exhibits a microhardness gradient along the depth direction that closely resembles the trend observed in C1. The cross-sectional microhardness profile demonstrates a characteristic gradual decrease from the surface (approximately 736 HV) to the core, reaching the base material hardness of 430 HV at the specified carburization depth of 0.6 mm.

It should be noted that the fluctuations in the curves in Figure 4 result from the use of a high-order polynomial for fitting. We employed a high-order polynomial primarily to obtain a highly accurate and continuous function to facilitate the subsequent integration calculations while the polynomial function exhibits some local fluctuations. In future work, we will explore the use of alternative fitting methods with a view to achieving both high accuracy and smoothness.

### 4.2. Results of Characterized Strength

**Figure 5:**
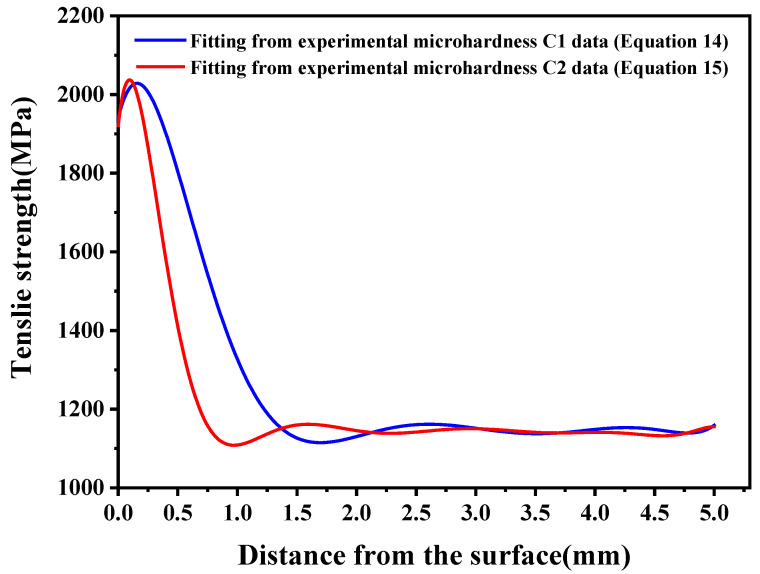
Strength distribution in the carburized layer.

By substituting the polynomial fit (Equation (12)) derived from experimental microhardness measurements of specimen C1 into Equation (6), the depth-dependent tensile strength profile of the carburized layer was obtained, as illustrated by the blue solid line in Figure 5. As shown in the following equation:

(14) $$\begin{array}{l} \sigma_{b}(x)=3.495x^{7}-69.411x^{6}+554.190x^{5}-2256.810x^{4}+4845.345x^{3}\\ \;\;\;-4911.759x^{2}+1221.126x+1940.468. \end{array}$$

Substituting the polynomial coefficients in Equation (14) into Equation (10), yields the equivalent tensile strength of 20CrNiMo steel carburized tensile specimen C1 in 1377.359 MPa.

Similarly, the through-thickness distribution of tensile strength in the carburized layer of specimen C2 was obtained. This distribution is mathematically represented by the following polynomial expression (shown as the red solid line in Figure 5):

(15) $$\begin{array}{l} \sigma_{b}(x)=-0.805x^{10}+22.542x^{9}-272.609x^{8}+1861.426x^{7}-7868.006x^{6}\\ \;\;\;+21198.130x^{5}-36009.633x^{4}+36450.923x^{3}\\ \;\;\;-18909.847x^{2}+2715.360x+1921.309. \end{array}$$

Substituting the polynomial coefficients in Equation (15) into Equation (10), yields the equivalent tensile strength of 20CrNiMo steel carburized tensile specimen C2 in 1279.834 MPa.

### 4.3. Results of Stress–Strain Curve

**Figure 6:**
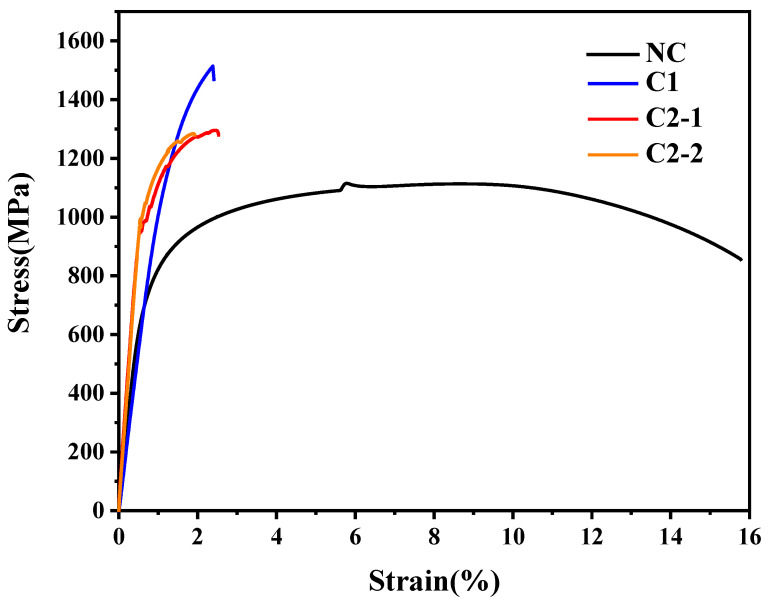
Stress–strain curve of tensile test specimen.

Figure 6 presents the comparative stress–strain curves of both heat-treated specimens. The NC specimen exhibits characteristic ductile material behavior, displaying four distinct deformation phases: (1) elastic deformation, (2) yield initiation at 781.4 MPa, (3) plastic deformation culminating in ultimate tensile strength of 1132.8 MPa, and (4) post-necking fracture with 54.6% reduction in area. Therefore, the basis for parameter selection in Equation (6) is the tensile strength value of the NC specimen.

The carburizing-quenching-tempering specimen exhibits characteristic brittle material behavior, demonstrating two distinct deformation stages: elastic deformation followed by limited plastic deformation. The mechanical properties are as follows: yield strength of 1318.3 MPa (68.7% enhancement compared to NC specimen), tensile strength of 1478.6 MPa (30.5% improvement relative to NC specimen), and reduction in area of merely 2.1%. For C1, the prediction error of the composite strength method was 6.847%.

Similarly, the C2 demonstrates a characteristic brittle material curve, primarily composed of elastic and a brief plastic deformation stage, with a tensile strength of 1295.3 MPa. For C2, the prediction error of the composite strength method was 1.194%.

### 4.4. Results of Fracture Surface Morphology

**Figure 7:**
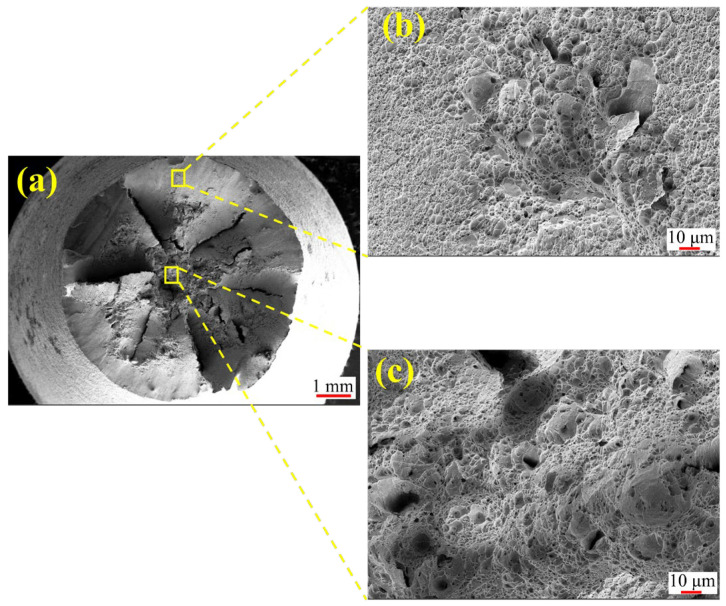
Fracture morphology of NC specimen: (**a**) Overall morphology; (**b**) Surface layer; (**c**) Core.

**Figure 8:**
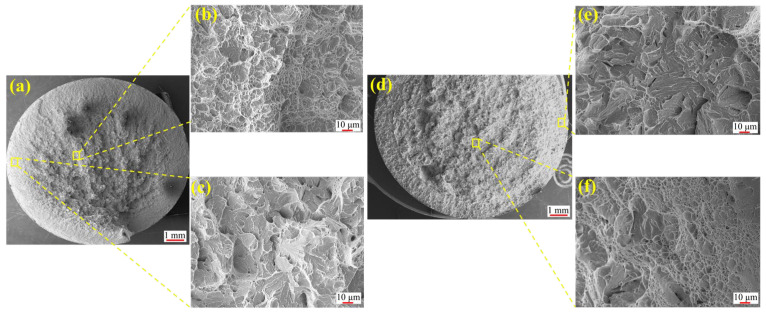
Fracture morphology of carburized tensile specimens: (**a**) Overall morphology of C1; (**b**) Core of C1; (**c**) Surface layer of C1; (**d**) Overall morphology of C2; (**e**) Surface layer of C2; (**f**) Core of C2.

Figure 7 presents the fracture morphology of the NC specimen. As a low-carbon alloy steel, 20CrNiMo exhibits significant necking during tensile testing (Figure 7a). The fracture surface shows dimples with varying contrast between the surface layer and the core, confirming a typical ductile fracture mechanism. In contrast, the carburized specimen (Figure 8) exhibits a mixed-mode fracture. The core region displays shallow dimples (Figure 8b,f), indicative of ductile fracture. Conversely, the surface layer reveals river-like patterns, characteristic of quasi-cleavage fracture (Figure 8c,e).

This phenomenon primarily originates from the formation of a carbon-enriched hardened layer on the surface during the carburizing process, which significantly enhances the strength and hardness of the surface region and results in a gradient distribution of strength and toughness from the surface to the core. Specifically, the surface exhibits high strength but poor ductility, while the core maintains relatively lower strength with superior toughness. During tensile loading, the performance mismatch between the hardened surface layer and the core matrix induces an uneven stress distribution, particularly promoting stress concentration at surface defects or microscopic notches. When the applied load exceeds the local strength limit of the surface layer, cracks tend to initiate at the surface and propagate rapidly along the hardened layer. As cracks extend into the core, the plastic deformation capacity of the core material imposes a certain degree of resistance to crack growth. However, continued crack propagation may ultimately lead to structural failure. Owing to the crack initiation at the high-strength but low-ductility surface, the fracture surface typically exhibits a mixed fracture mode characterized by brittle features in the surface layer and ductile tearing features in the core region.

### 4.5. The Proposed Composite Strength Method vs. Conventional Multilayer Method

**Figure 9:**
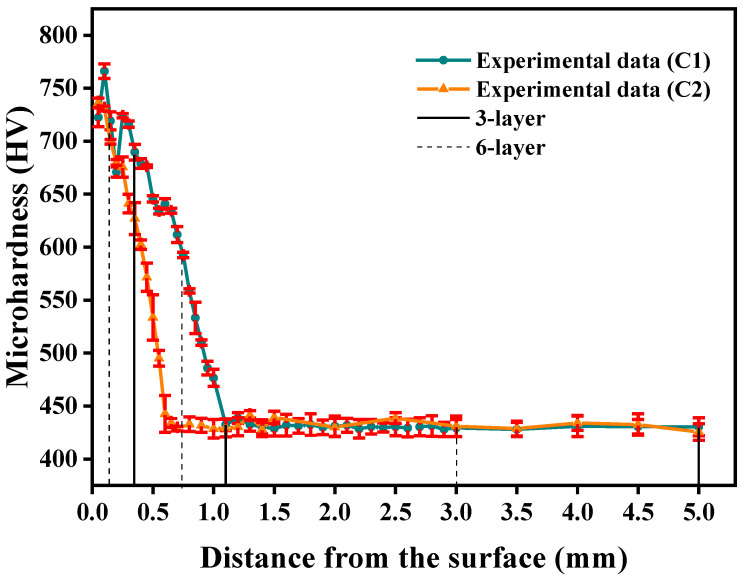
Application of multilayer method with Voigt on carburized specimens.

The C1 and C2 specimens were divided into three layers [39] using the conventional Voigt-based multilayer method, as illustrated in Figure 9. It is important to note that the specific number of layers was designed according to the hardness and strength distribution within the carburized layer: the surface region with oscillatory HV, the subsurface region with approximately linear HV, and the core region with stable HV. Based on Equation (11), the resulting equivalent strengths are as follows:

(16) $$\begin{array}{l} \bar{\sigma }=\frac{2\sum_{i=1}^{3}\sigma_{i}\int_{x_{i-1}}^{x_{i}}(R-x)dx}{R^{2}}\\ \;\;=\frac{\sigma_{1}(2R\cdot x_{1}-x_{1}^{2})+\sigma_{2}(2R\cdot x_{2}-2R\cdot x_{1}-x_{2}^{2}+x_{1}^{2})+\sigma_{3}(R^{2}+x_{2}^{2}-2R\cdot x_{2})}{R^{2}}, \end{array}$$

where *σ_i_* is defined as the mean value of the strength over the entire *i*-th layer. When the number of layers was increased to six (the six-layer division was implemented by further subdividing the midpoints of an initial three-layer partition), the calculated equivalent strengths were as follows:

(17) $$\bar{\sigma }=\frac{2\sum_{i=1}^{6}\sigma_{i}\int_{x_{i-1}}^{x_{i}}(R-x)dx}{R^{2}}.$$

**Figure 10:**
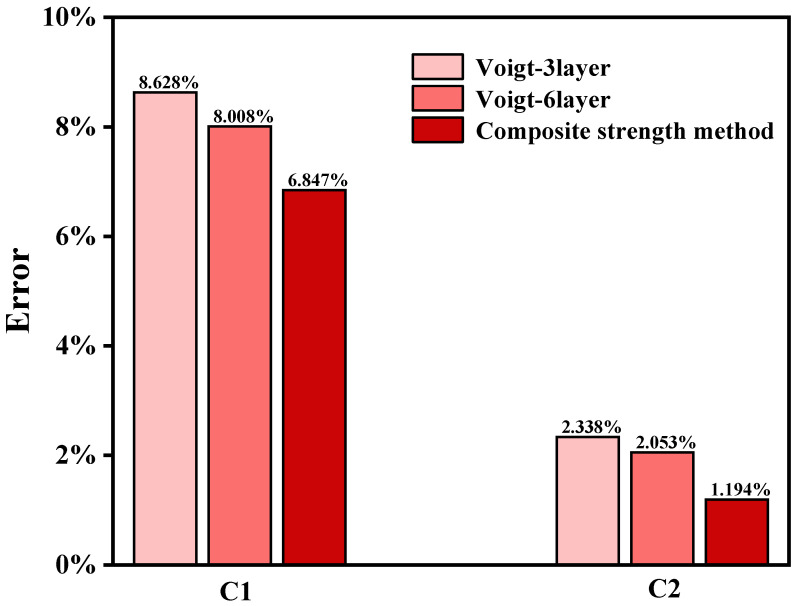
Prediction errors obtained by different methods for the carburized specimens.

A comparison of the errors between the composite strength method and the conventional multilayer method is presented in Figure 10. Specifically, the error is calculated using the following formula:

(18) $$Error(\%)=\frac{\sigma_{\mathrm{experimental}}-{\bar{\sigma }}_{\mathrm{predicted}}}{\sigma_{\mathrm{experimental}}}\times 100\%,$$

where $\bar{\sigma }$_predicted_ represents the predicted equivalent tensile strength, namely the results calculated by the composite strength method proposed in this study (Section 4.2) and those obtained from the conventional multi-layer method using Equations (16) and (17), *σ*_experimental_ indicates the tensile strength values directly measured from tensile tests (Section 4.3, with Sample C1 being 1478.6 MPa and Sample C2 being 1295.3 MPa). The error is therefore determined by comparing the predicted strength values against the experimental measurements.

As can be seen from Figure 10, the error of the multilayer method decreases as the number of layers increases, which is attributed to the improved prediction accuracy with finer discretization. However, the predictive accuracy of the multilayer method still lags behind that of the proposed composite strength method. For specimen C1, the proposed method improves prediction accuracy by over 13.2%, while for specimen C2, the enhancement exceeds 41.8%. In summary, by establishing an equivalent model for the strength gradient of the carburized layer, this method provides critical computational support for optimizing the carburizing process of key transmission components. It also demonstrates significant practical value for strength assessment and design optimization in engineering applications.

## 5. Conclusions and Outlook

A mechanical model for predicting the tensile strength of carburized specimens via 20CrNiMo steel was established, incorporating quantitative characterization of the mechanical properties in the graded carburization layer. The model’s reliability was systematically validated through tensile testing, yielding the following principal conclusions:

- A novel composite strength method for the quantitative strength prediction of carburized materials is established. By utilizing only microhardness data from the carburized layer, this approach provides accurate tensile strength predictions without uniaxial tensile testing, demonstrating significant potential for engineering performance evaluation.
- Experimental validation using C1 and C2 specimens with different carburizing depths confirms that the tensile strength prediction error of this method remains below 7%, with a minimum error reaching 1.2%. Compared to the conventional Voigt-related multilayer method, the proposed method demonstrates a significant improvement in both evaluation efficiency and accuracy, achieving over 41.8% enhancement in prediction precision.
- By introducing the Taylor series expansion, a mapping relationship between discrete hardness values and continuous strength properties has been established. This approach breaks through the Voigt theory-based layered discretization approximation paradigm. Requiring only hardness as the core measurement metric, it transcends the traditional research path that relies on complex crystal structure-microstructure-mechanical property correlations, demonstrating significant potential for engineering applications.
- Due to experimental limitations, this study validates the proposed model only on 20CrNiMo steel. However, future research could extend the verification to different material types, specimen diameters, and complex testing conditions (irregularly shaped specimens) to further demonstrate the universality of the model. The authors will continue in-depth research in this field with the aim of achieving more valuable results.

## Figures and Tables

**Table 1:** Chemical composition of 20CrNiMo steel (wt.%).

Element wt%						
C	Si	Mn	Cr	Ni	Mo	Fe
0.219	0.27	0.60	0.58	0.53	0.24	Balance

**Figure A1:**
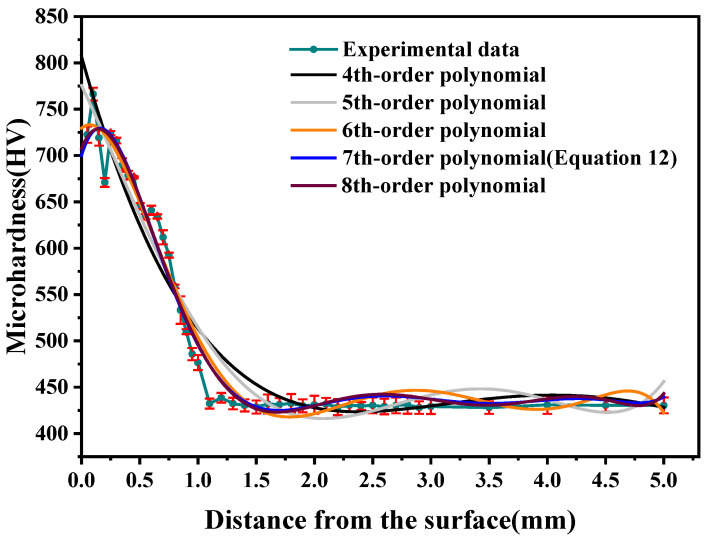
Microhardness fitting results for Taylor series expansion of different degrees.

## Data Availability

The original contributions presented in the study are included in the article; further inquiries can be to the corresponding author.

## Appendix A

### Appendix A.1

It is worth noting that the specific polynomial coefficients selected in this study were derived from fitting results. For instance, the coefficients corresponding to Equation (12) belong to the seventh order term. In other words, the polynomial order is not inherently fixed, the objective is to effectively represent the fitted data. For clarity, the process of determining the optimal polynomial order is illustrated in Figure A1.

For Equation (12) in Figure 4, polynomial orders of 4-8 were evaluated, yielding coefficients of determination (R^2^) of 0.951746, 0.961467, 0.976755, 0.981290 and 0.981513, respectively. The 7th-order polynomial was selected owing to its superior fit, as it achieves an optimal balance by effectively capturing the underlying data trends without exhibiting underfitting or overfitting.
